# Applications and implicit assumptions in dementia risk scores: A scoping review of the LIBRA score

**DOI:** 10.1177/25424823261416457

**Published:** 2026-01-22

**Authors:** Wouter MR Kant, Wieske K de Swart, Jim M Smit, Marco Loog, Jesse H Krijthe

**Affiliations:** 1 Institute for Computing and Information Sciences, 541121 Radboud University, Nijmegen, Gelderland, The Netherlands; 2 Pattern Recognition Laboratory, 225112 Delft University of Technology, Delft, Zuid-Holland, The Netherlands

**Keywords:** Alzheimer's disease, causality, cognitive decline, dementia, risk assessment, risk factors, risk score

## Abstract

Dementia risk scores are commonly used tools to estimate the risk of developing Alzheimer's disease and dementia. We lack an overview of what risk scores are used for, what is claimed they ought to be used for, and whether they are suitable for these applications. To address this, we use the ‘Lifestyle for Brain Health’ (LIBRA) score as a representative example risk score and conduct a literature review to study its applications. The goals of this study are (1) to create an overview of how the LIBRA score has been utilized in scientific articles, (2) to record other applications that these same articles mention, and (3) to critically assess whether LIBRA is suitable for these applications. Of the 66 articles included in our review, 36 involved analyzing associations of LIBRA with dementia, cognition, or other outcomes. We also identified several other applications, with 32 articles mentioning LIBRA as an estimate of ‘dementia prevention potential’, 6 articles used LIBRA as a surrogate outcome for their trial or intervention, and 7 articles mentioned that it could help support clinician decisions in practice. Although there is a clear need for tools that can be used for these applications, the amount of evidence supporting the suitability of dementia risk scores for many of these applications is limited. We recommend that researchers transparently report the purposes of these dementia risk scores, which may include causal tasks, and that research is done to evaluate whether it is valid to use these scores in this way.

## Introduction

Dementia is a group of disorders, most commonly caused by Alzheimer's disease, with at least 50 million people suffering from it worldwide, with that number set to increase threefold by 2050.^1^ Many factors have been shown to impact the risk of dementia. These factors include non-modifiable factors such as age and sex, as well as modifiable factors such as alcohol usage and physical activity.^2^ Multiple risk scores have been developed in order to estimate which people are at a higher risk of dementia than others. These scores include, among others, the Cardiovascular Risk Factors, Aging, and Dementia (CAIDE) score, the Australian National University Alzheimer's Disease Risk Index (ANU-ADRI) score as well as the Lifestyle for BRAin health (LIBRA) score^3–5^ These scores have been shown to be predictive of dementia risk across multiple populations^6–8^ They have also been shown to predict other dementia-related ailments such as mild cognitive impairment and neurovascular pathology.^9,10^

Due to their ease of use, they have also been implemented into risk assessment tools, that may be utilized by individuals to quickly assess their relative dementia risk.^11^ However, since their introduction, they have also been utilized in more ways than simply dementia risk prediction. Additional applications of dementia risk scores include being used as a surrogate outcome measure for dementia, estimating the potential effect that an intervention would have on dementia risk, as an inclusion criterion for studies, and for variable selection^11–14^ However, each of these applications requires different assumptions about the data and/or the dementia risk score in order to be valid. In order to interpret the results of utilizing dementia risk scores in these ways, careful assessment of the underlying assumptions is necessary.

In contrast to other dementia risk scores, the LIBRA score is explicitly constructed with these additional applications in mind. Namely, it is constructed with the goal of estimating the ‘potential for dementia prevention’ and ‘room for improvement’ of individuals.^5,15^ Similarly to CAIDE and ANU-ADRI, it is based on results of prospective cohort studies, and optimized to predict dementia risk. LIBRA differs from CAIDE and ANU-ADRI in the selection of risk factors that it includes, because it specifically only includes modifiable risk factors.^5^ Although leaving out important non-modifiable predictors such as age worsens its performance in predicting dementia risk, this is done with the goal of improving its capability of estimating the ‘dementia prevention potential’ of individuals.^5^ However, given that the LIBRA score is based on observational data, it is not self-evident that the score will be optimal for estimating the potential for preventing dementia. While the ability to estimate dementia risk has been validated across multiple populations, the suitability of the LIBRA score for other applications has, so far, not been extensively studied. The purpose of this review is to discuss the suitability of different applications of the LIBRA score. Although several of these applications may also apply to other dementia risk scores, we limit our scope to LIBRA because it was explicitly designed to reflect dementia prevention and therefore most directly implies a potential for causal interpretations.

We conducted a scoping review to answer the question: ‘How is the LIBRA risk score used and discussed in scientific literature?’ For each of the identified applications, we include a critical evaluation to discuss how often it is used, what assumptions underlie it, and whether additional evidence is needed to verify whether the score is suitable for this application. In the Background section, we describe how the LIBRA score is constructed as well as explain the causal assumptions that are relevant to estimate dementia prevention potential. Then, in the Methods section, we describe our literature search and how we scored the articles in terms of which applications and assumptions of the LIBRA score were reported. We then show the results of this search in the Results section. In the section Applications of Risk Scores, we discuss the results of our search, and critically evaluate each application. Finally, in the Discussion, we summarize our findings about the applications of the LIBRA score, relate them to other dementia risk scores, and give recommendations for future research.

## Background

### Dementia risk scores

Dementia risk scores estimate the risk of developing dementia based on a set of risk factors. Several models have been developed using different risk factors and scoring systems. To be useful in practice, they should also be extensively validated and be available as accessible tools.

A systematic review by Hou et al.^16^ identified 61 dementia risk models: 4 for mid life risk, 39 for late life risk and 18 for specific patient groups. The most commonly included predictors were cognitive measures, followed by demographics, genetics and health and lifestyle factors. Only eight models were externally validated. A broader discussion on dementia risk scores is provided by Anstey et al.^11^ They highlight three dementia risk scores that contain modifiable risk factors, are available as practical tools and have been extensively validated: CAIDE, ANU-ADRI and LIBRA.

The CAIDE score^3^ is based on a study of 1409 subjects in midlife with a follow-up of 20 years. Included factors are age, education, sex, systolic blood pressure, body-mass index, cholesterol, physical activity and APOE ε4 status. Weights for the individual risk factors were based on regression coefficients and the total score is given by the sum of the individual weights. The ANU-ADRI^4^ includes 11 risk factors and 4 protective factors for Alzheimer's disease and can be computed without clinical assessment. The factors were identified through systematic reviews, and the weights are derived from the odds ratio found in existing meta-analyses or calculated from cohort studies. The LIBRA score^5^ differs from other dementia risk scores by focusing on prevention and exclusively using modifiable risk factors. The construction of LIBRA will be described below.

### LIBRA score

The construction of the LIBRA risk index was done in two parts. Firstly, in Deckers et al.^17^ modifiable risk factors for dementia were identified by a systematic review and Delphi consensus study. The researchers identified fourteen risk and protective factors based on expert opinions and the results from 291 studies identified in their systematic review. For each risk factor, the relative risk is extracted from existing meta-analyses. The studies identified in the meta-analyses can differ in terms of used cut-offs, adjustment and reported effect estimate. Although these reported effect estimates, odds ratio (OR), relative risk (RR) and hazard ratio (HR), are different,^18^ they are often considered the same effect size measure under the assumption that dementia is a rare event.^19^

The results of this study are used to create the LIBRA score, which is described in Schiepers et al.^5^ For each risk factor, the natural logarithm of the relative risk is computed, and standardized by setting the minimum to 1 (Table 1). A binary scoring approach is used, where each factor is counted as present or absent based on available (proxy) measures and a set threshold. The total LIBRA score for an individual is the sum of the scores assigned to all the factors they are exposed to.

**Table 1.** List of modifiable risk factors in the LIBRA risk index, the relative risk extracted from a meta-analysis for each factor and the corresponding weight in the LIBRA score.

Modifiable risk factor	Relative risk (RR)	Score
Low/ moderate alcohol consumption	0.74	−1.0
Coronary heart disease	1.36	+1.0
Physical inactivity	1.39	+1.1
Renal dysfunction	1.39	+1.1
Diabetes	1.47	+1.3
High cholesterol	1.54	+1.4
Smoking	1.59	+1.5
Obesity (midlife)	1.60	+1.6
Hypertension (midlife)	1.61	+1.6
Mediterranean diet	0.60	−1.7
Depression	1.85	+2.1
High cognitive activity	0.38	−3.2

Several assumptions are made in this model. Deckers et al.^17^ note that ‘causality between factors and outcome cannot be demonstrated’, and Schiepers et al.^5^ note that ‘our risk prediction algorithm assumes additivity of risk factor effects and possible interactions between risk factors were not modeled in our algorithm’. Given that the LIBRA score is calculated as the sum of the logarithm of the relative risk of each factor, this algorithm assumes the relative risk to be multiplicative.

### Risk prediction versus prevention potential

As stated earlier, the LIBRA score differs from the ANU-ADRI and CAIDE scores in its goal to estimate the ‘prevention potential’ of individuals. Prevention of dementia is a separate task from dementia prediction, because the association between a risk factor and dementia risk does not necessarily equate to the reduction in risk that is seen after an intervention on this risk factor.^11^ In order to assess whether a risk score can perform this causal task, we need to consider several causal assumptions.

The first of these assumptions is conditional exchangeability, also referred to as ‘no missing confounders’. This assumption states that the group that is exposed to the risk factor at the start of a study must be similar to the group that is unexposed to the risk factor. This means that all confounding variables must be found and adjusted for. If this is not the case, then it is possible that a confounding variable is responsible for the association, rather than it being a causal link.

A second causal assumption that may be examined is consistency, which means that the hypothetical intervention of which we are estimating the effect must be well-defined and corresponding to the intervention in the observed data. Useful questions to ask are: How is the intervention achieved (e.g., how will the person achieve higher physical activity?), by how much will the risk factor be reduced (e.g., will they take a walk or run a marathon?), and which life phase the individual is in (e.g., at which age will the physical activity be increased?). This helps us to be precise about what the exact question is that we are asking.

In the ‘Applications of Risk Scores’ section, we will discuss the assumptions that are mentioned above in order to assess whether the score is suitable for applications related to estimating dementia prevention.

## Methods

### Database search strategy

**Figure 1.**
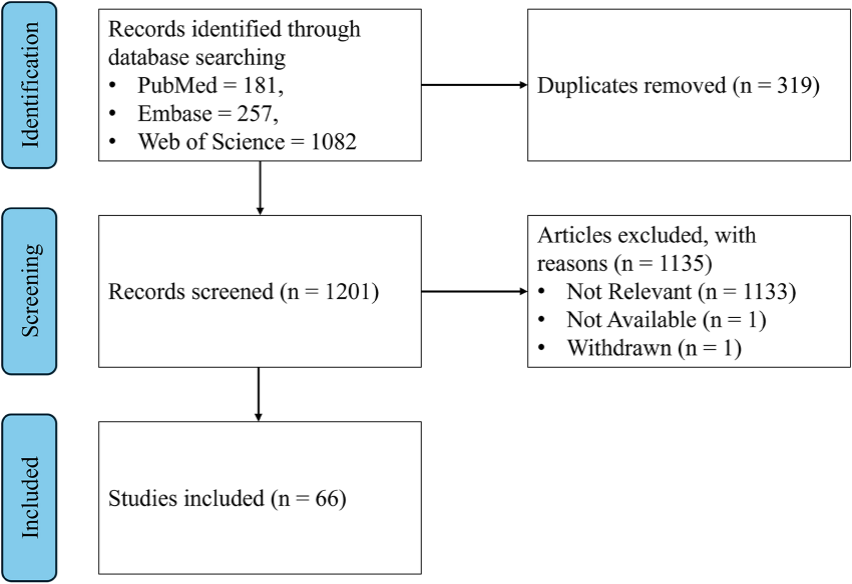
Flow diagram depicting the steps of the literature search.

We conducted a scoping review following the framework described by Arksey and O’Malley^20^ and Peters et al.^21^ The search strategy and inclusion criteria were discussed with a librarian and updated accordingly. Two authors separately searched in the PubMed, Embase and Web of Science databases. For PubMed and Embase both researchers used the query “LIBRA”. For Web of Science a different approach was taken due to the larger number of results. One researcher scanned all results based on the query “LIBRA” and another researcher scanned all results for a more restrictive query (TS = (LIBRA) AND (TS = (dementia) OR TS = (cognition) OR TS = (cognitive))). This approach was validated by a scientific librarian. With this approach, we could spend additional attention to the records most likely to be of interest, while simultaneously making sure that no publications where missed that were not retrieved by this second query. The flow diagram is shown in Figure 1.

The search with the query “LIBRA” was performed on March 18, 2025 and retrieved 181 publications from PubMed, 257 from Embase and 1082 from Web of Science. The second query in Web of Science retrieved 73 results on March 31, 2025. After removing duplicates, this resulted in 1202 publications that were checked for inclusion. Our inclusion criteria were as follows: (1) must mention the Lifestyle for Brain Health (LIBRA) risk score, (2) English language and (3) published in a peer-reviewed journal. Most publications were excluded for the first reason, due to the existence of several other tools with the same name. Usually, this was immediately clear from title and/or abstract, but in some cases the full text was checked to confirm. One publication was excluded because it was withdrawn and one because the full text was not available. The final inclusion consisted of 66 publications^5,8,10–15^^,22–79^

### Scoring items

Two researchers independently scored all included publications for items regarding the application of LIBRA in the paper, the mentioned potential applications of LIBRA and the assumptions that the publication mentions about these applications of LIBRA. Subsequently, disagreements between the researchers were discussed, and consensus was reached about all scoring criteria. The following sections were scored in all articles.

*Applications of risk score in the article.* We recorded the applications of LIBRA in 9 different categories, which were added and adapted based on the identified literature. We also included planned applications when these were clearly described in a published protocol. ‘*Dementia prediction*’ was scored when the association between the LIBRA score and a dementia outcome was analyzed. ‘*Association cognition*’ was scored when the effectiveness of LIBRA at predicting any measure of cognition that is not dementia was measured. This includes effectiveness at cognitive tests, as well as mild cognitive impairment. ‘*Association other*’ was scored when the association of LIBRA and a non-cognitive outcome was analyzed, which includes cardiovascular outcomes, vascular damage and brain mass. ‘*Adjust for lifestyle*’ was scored when the LIBRA score is incorporated into a prediction model as a simple way to adjust for all modifiable factors that LIBRA includes. ‘*Surrogate outcome*’ was scored when LIBRA was used as a surrogate outcome in longitudinal trials, to substitute the ‘true’ dementia outcome, which would take a long time to measure. ‘*Targeting subgroups (HTE)*’ is scored for articles that study heterogeneity of treatment effects (HTE) in relation to the LIBRA score, which would indicate LIBRA's potential for identifying subgroups that would benefit more from an intervention. ‘*Inclusion criteria*’ was scored when the LIBRA score is used as one of the inclusion criteria for a trial or intervention. ‘*Motivational tool/ awareness*’ was scored when the LIBRA score was involved in research on awareness of dementia or motivation for lifestyle changes. Finally, ‘*Variable selection*’ was scored when the LIBRA score was explicitly used to inspire which variables should be included in the study. This includes machine learning prediction models that required variable selection, but also studies that examined the awareness of risk factors.

*Mentioned applications of risk scores.* Apart from the ways in which LIBRA was used in the articles themselves, we recorded all the applications of the LIBRA score that were mentioned. All categories of the previous section were included in this analysis, except for ‘*Variable selection*’. Application of the LIBRA score in a category was not immediately counted as a mention, only when this application is discussed in broader terms, connected to related work, or linked to a practical implementation. There were two additional categories for mentioned applications of risk scores: ‘*Supporting clinician decisions*’ was scored if it was mentioned that the LIBRA score may be used in practice to support practitioners in any way. ‘*Prevention potential*’ was scored if it was mentioned that it is literally an indication of ‘prevention potential’, or ‘room for improvement’ in terms of improving dementia risk.

*Mention of assumptions.* There are multiple assumptions that underlie the applications of the LIBRA score. We divide these into two categories. The first category ‘*Modeling assumptions*’ was scored when any assumption was made that is necessary for predictive applications of LIBRA. These include a lack of interactions and the multiplication of relative risks in the construction of the LIBRA score. The second category ‘*Causal assumptions*’ consists of assumptions that are required when utilizing the LIBRA score for more than simply predictive tasks, as well as any mention that a change in the LIBRA score might not equal a change in dementia risk.

## Results

**Figure 2.**
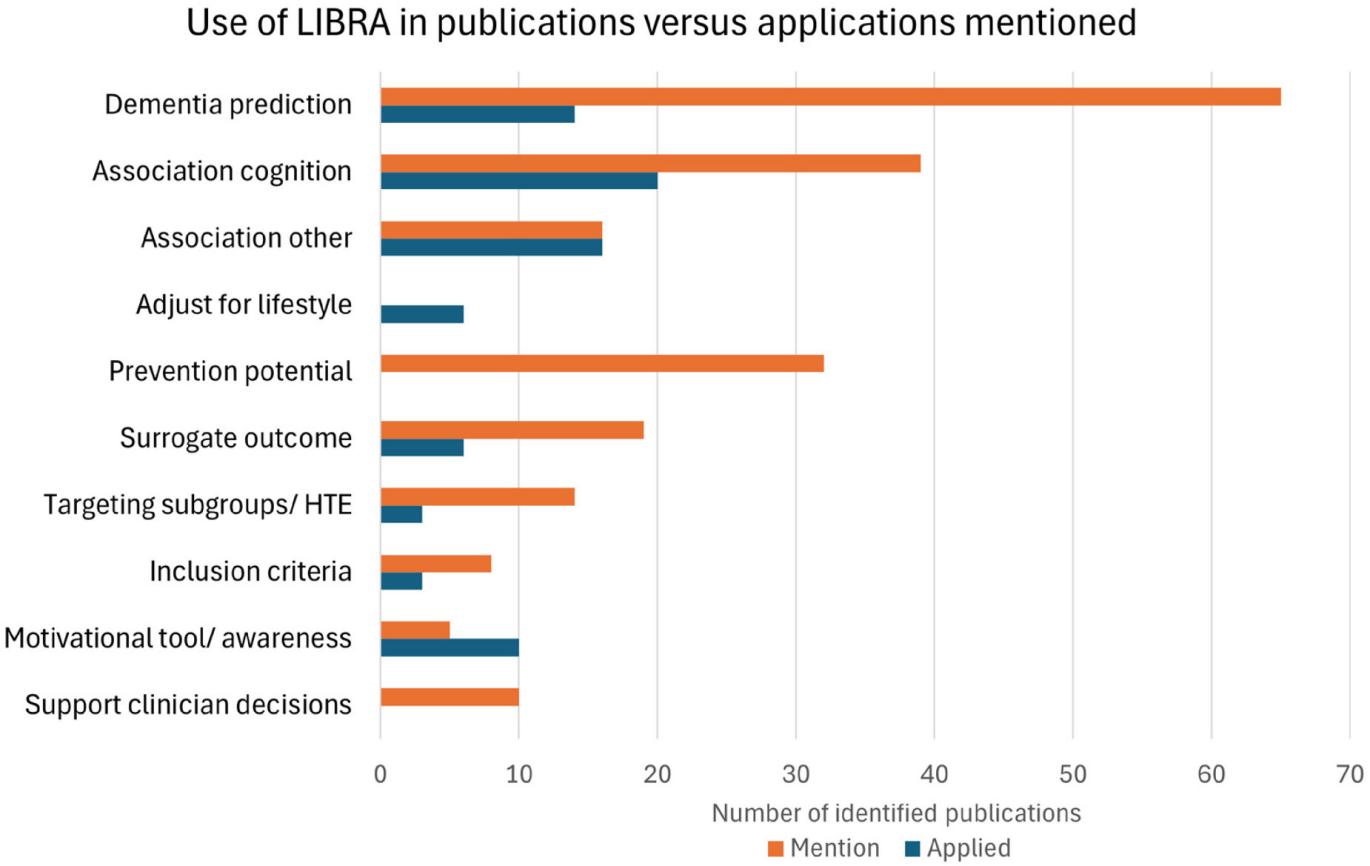
Number of publications using LIBRA for different purposes versus the number of publications mentioning that potential application of LIBRA. See the ‘Applications of risk scores in the article’ subsection of the Methods section for explanations of the categories.

In Figure 2, we show the number of publications that used LIBRA for each purpose, as well as the number of publications that mentioned each application. Interestingly, the applications that are most often mentioned about LIBRA do not always correspond to the ways LIBRA is most often used in research.

Almost all included publications (65 of 66) mentioned the use of LIBRA as a dementia risk score and/or the use of LIBRA for the prediction of dementia. We found 39 studies that mentioned an association of LIBRA with cognitive outcomes, usually by referring to earlier work. 16 studies also mentioned an association of LIBRA with other outcomes, such as brain biomarkers. Of the publications that studied associations related to LIBRA, we found 20 studies involving the association between LIBRA and cognition, 13 studies that involved the association of LIBRA and dementia, and 16 studies that involved associations with other predictors or outcomes. In 14 studies the LIBRA index was used for variable selection, usually either to select variables included in a (machine learning) model or to select risk factors included in a questionnaire. We also found 6 studies in which LIBRA was used as a covariate to adjust for differences in lifestyle. This use case of LIBRA was not mentioned in the rest of the literature.

LIBRA is also often proposed for other use cases. The most popular mention was the use of LIBRA as a measure of ‘prevention potential’ or ‘room for improvement’, which was mentioned in 32 publications. The second most popular mention was the use as a surrogate outcome in a trial or intervention study, which was mentioned in 19 publications. In 6 studies LIBRA was used or proposed as the outcome of a trial or intervention, as a surrogate for dementia risk. 14 publications mentioned the use of LIBRA as a tool to target subgroups or select individuals in practice, while only 3 studies investigated this possibility by examining heterogeneous treatment effects related to LIBRA. 8 publications mentioned the use of LIBRA as inclusion criteria for trials or interventions. In 5 studies the use of LIBRA as a motivational tool was mentioned. While this was not often mentioned, we did find 10 publications using LIBRA in an awareness study or motivational campaign. Finally, 10 studies mentioned that the LIBRA score could be used to support clinician decisions.

Not many papers have mentioned any assumptions that underlie the LIBRA score. In total, 11 papers mention any modeling assumptions. Of these, 8 articles mentioned that interactions between modifiable risk factors are not taken into account in the construction of the LIBRA score.^5,8,32,42,45,58,63,75^ Two papers mention that no interactions with non-modifiable factors (such as age) were taken into account.^62,65^ A few articles mentioned other modeling assumptions such as risk factor history not being taken into account^63^ or the additivity assumption of the effects of the risk factors in LIBRA.^5^ Five papers in total have hinted at the fact that the LIBRA score may not be causally interpretable. These include that ‘risk prediction is not risk reduction’,^11,12,15,62^ and one paper mentions that there might be (confounding) factors missing in LIBRA.^63^ Papers that do a cross-sectional analysis mention that the conclusions may not be causally interpreted, or mention the possibility of reverse causation.^40,41,69^

## Applications of risk scores

In this section, we discuss the different applications that were either encountered or mentioned in the included papers. For each application, we first discuss the statistics of how often this was found, and then discuss potential implications and dangers of using the LIBRA score for this purpose.

### Dementia prediction

We identified 13 publications that examined the association between the LIBRA score and dementia. Given that LIBRA is a dementia risk score, using (modifiable) risk factors for dementia, one would anticipate it to be predictive of dementia risk. However, validation of this claim in both the general population or specific subgroups is important. In addition, these studies can help to determine how predictive LIBRA is and help identify factors that might influence the association with dementia.

In Table 2 we summarized the results of the examined associations between LIBRA and dementia. This includes a wide variety of studies, with different populations and varying results. Most studies used Cox proportional hazard models, often either with age as the timescale or corrected for age and sometimes other variables such as sex and education. Not all studies adjusted for age: notably, two studies in late life that did not find significant associations did not adjust for age or correct for mortality.^30,75^ Not all LIBRA factors are always included, with the number of factors ranging from 7 to 12. Given the wide variety in results it is difficult to conclude what the predictive value of the LIBRA score is. The meta-analysis by Van Asbroek et al.^72^ combined data from 21 prospective cohorts across six continents and found a hazard ratio of 1.06, meaning one point increase in LIBRA was associated with 6% increase in dementia risk.

**Table 2.** Results from 13 publications that studied the association between LIBRA score and dementia. Shown are the name of the dataset or study, the number of subjects, the age at baseline (reported as mean ± standard deviation) and the reported result. Results show the hazard ratio for a one point increase in LIBRA (except PATH, which reported the HR per standard deviation increase in LIBRA) or the AUC. The CC75C study reported a group comparison, with mean LIBRA in the group with incident dementia −0.93±2.2 and no incident dementia −0.51±2.2 .

Study	Region	Subjects	Age	Result
LIBRA (only modifiable risk factors)				
CHS-CS^47^	USA	3375	74.8±4.9	AUC=0.51 (0.48–0.54)
HRS-ADAMS^47^	USA	548	79.5±6.3	AUC=0.52 (0.45–0.59)
MAP^47^	USA	2184	80.0±7.6	AUC=0.53 (0.48–0.57)
DESCRIPA^75^	Europe	1811	83.2±2.5	HR = 0.93 (0.88–0.99)
preDIVA^53^	Netherlands	3274	74.3±2.5	HR = 1.02 (0.96–1.09)
CAIDE latelife^8^	Finland	604	69.8±3.4	HR = 1.03 (0.84–1.24)
Meta-analysis^72^	Worldwide	31680	71 (median)	HR = 1.06 (1.04–1.08)
MAAS^63^	Netherlands	1417	50.4±15.8	HR = 1.08 (0.99–1.16)
DESCRIPA^75^	Europe	4320	74.5±2.9	HR = 1.08 (1.03–1.13)
ELSA^42^	UK	7460	65.7±9.4	HR = 1.09 (1.04–1.14)
3C Study (ε 4− )^54^	France	4131	73.9±5	HR = 1.09 (1.05–1.13)
DESCRIPA^75^	Europe	3256	65.0±4.0	HR = 1.10 (1.02–1.18)
ELSA^63^	UK	7587	65.9±8.9	HR = 1.13 (1.09–1.16)
ELSA^32^	UK	6364	64.9±8.6	HR = 1.13, 1.07–1.19
3C Study (ε 4+ )^54^	France	1039	73.9±5	HR = 1.15 (1.08–1.22)
MAAS^5^	Netherlands	949	65.0±8.7	HR = 1.19 (1.08–1.32)
CAIDE midlife^8^	Finland	1024	47.8±4.7	HR = 1.27 (1.13–1.43)
PATH^15^	Australia	2409	62.5±1.5	HR = 1.29 (1.11–1.49) per sd*
CC75C^30^	UK	278	87.9±3.2	p-value 0.154*
LIBRA + age, sex, education				
HCS^41^	Australia	3306	63.8±7.8	AUC = 0.65 (0.60–0.71)
MAP^47^	USA	2184	80.0±7.6	AUC=0.65 (0.61–0.69)
HRS-ADAMS^47^	USA	548	79.5±6.3	AUC=0.67 (0.61–0.74)
CHS-CS^47^	USA	3375	74.8±4.9	AUC=0.70 (0.68–0.73)
preDIVA^53^	Netherlands	3274	74.3±2.5	HR = 1.07 (1.02–1.12)
DESCRIPA^75^	Europe	1811	83.2±2.5	HR = 1.08 (1.03–1.17)
DESCRIPA^75^	Europe	3256	65.0±4.0	HR = 1.11 (1.04–1.17)
DESCRIPA^75^	Europe	4320	74.5±2.9	HR = 1.14 (1.10–1.17)
MAAS^5^	Netherlands	949	65.0±8.7	HR = 1.25 (1.19–1.32)

The scores in the LIBRA model are based on relative risk measures derived from meta-analyses,^17^ as discussed in the Background section, which allows us to compute the theoretical predictive ability of the LIBRA index. If we assume all modeling assumptions are correct, we would expect a 1 point increase in LIBRA to be associated with a 35% increase in dementia risk. The results that we identified in our review, shown in Table 2, are far removed from this theoretical 35%, which might be an indication that not all modeling assumptions are correct. For instance, overlap or interactions between risk factors could change the effect on the total score.

Another point of discussion is that the choices to exclude non-modifiable factors from the LIBRA score and only use binary cut-offs for the included factors might diminish the potential for dementia prediction. The use of binary factors ignores detailed information in the data,^37^ especially for continuous risk factors, such as physical and cognitive activity. In addition, for some risk factors the optimal threshold is unclear^17,62^ and studies often use different thresholds depending on data availability. While the use of binary factors makes the index easy to compute, this should not be an issue with the use of modern technologies.^80^ Coley et al.^12^ propose a continuous version of the LIBRA score based on z-scores of the individual factors, but also mention that further investigation is required to determine the best format.

For the exclusion of non-modifiable risk factors, there is a lot of evidence that this decreases the predictive ability, with several studies showing increased hazard ratios and especially better discriminative performance when the factors age, sex, and education were added to the LIBRA index.^5,47,53,75^ The authors purposefully avoided non-modifiable factors, reasoning that this would ‘increase its potential application in the development of tailored interventions and primary prevention’.^5^ They suggest that a model with additional scores for education, age and sex ‘can be considered a risk-prediction model’, while LIBRA itself ‘may be regarded as a model for dementia prevention’. However, this last claim implies that the LIBRA score is a causal model, for which either additional evidence or strong assumptions are needed. This claim will be further discussed in the Subsection ‘Prevention Potential’.

### Associations

We identified 29 studies that examined associations between LIBRA and factors other than dementia. 20 of these involved associations between LIBRA and cognition and 16 involved associations with other factors. Associations with cognition were often determined based on measures of cognitive functioning derived from cognitive test results, usually covering multiple domains, such as information processing speed, executive functioning and verbal memory. Some studies defined groups based on markers for (mild) cognitive impairment (MCI)^10,26,44^ or cognitive decline.^28^ In addition, several studies examined incident MCI^5,8,30^ or rate of cognitive decline.^15,46,61^ Associations with other factors included brain biomarkers,^29,35,44,56,65^ socioeconomic factors,^46,57,58,67^ genetic factors,^29,52^ depression,^40,42^ retinal measures,^24^ self-reflection,^36^ medication usage^38^ and parental family history of dementia.^77^

In 5 studies LIBRA was used in a mediation analysis using structural equation modeling. Three studies examined the mediation by LIBRA on another association, namely the association between wealth and dementia,^32^ the association between depressive symptoms and dementia^42^ and the association between socioeconomic status and cognitive functioning.^59^ Two other studies examined the mediation by ‘Brain Age Gap’^35^ or MRI markers^44^ on the association between LIBRA and cognition.

While for associations with cognition the LIBRA score is generally used as a predictor, for other associations it was sometimes used as the outcome. We identified 5 out of 16 publications that used LIBRA as the outcome, for the association with measures of retinal health,^24^ disadvantages neighborhoods,^57^ social determinants,^58^ sociodemographic factors^67^ and parental family history.^77^ In these studies LIBRA was, often explicitly, used as a (surrogate) measure for dementia risk. The study by Barrett et al.^24^ was the only one that mentioned the use of an indirect measure as a limitation.

### Adjust for lifestyle

We identified 6 studies that used the LIBRA risk score as a covariate to adjust for the effect of lifestyle factors on dementia risk. LIBRA was used as a covariate for the association between self-reflection and AD-sensitive markers,^36^ cognitive and social activities and dementia,^39^ menopausal hormone therapy and cerebrospinal fluid biomarkers,^48^ sleep and cognitive performance,^49^ area-based socioeconomic deprivation and cognitive functioning^60^ and finally ambient air pollution and cognitive functioning.^69^

Demnitz-King et al.^36^ added LIBRA as a covariate to test if the association is independent of possible lifestyle pathways.^36^ And Jauregi-Zinkunegi et al.^48^ tried to avoid overfitting the model by using this composite measure instead of entering each factor separately.

While several studies mentioned LIBRA being a score to measure lifestyle quality or lifestyle-related dementia risk, we did not find any studies that discussed the potential of LIBRA as a confounder. Du et al.^38^ did mention advantages of the use of composite scores in general, namely that this ‘reduces the overall number of tests thereby reducing the probability of spurious or irreproducible findings’.^81^

### Prevention potential

We have found that 32 papers in total mention that the LIBRA score is a measure of ‘prevention potential’ or the ‘room of improvement’ for patients in terms of their dementia risk.

As explained in the Background section, the task of estimating prevention potential is different from the task of dementia prediction because of the causal nature of the task, and it can be helpful to examine the assumptions of ‘conditional exchangeability’ and ‘consistency’. There are arguments for and against LIBRA fulfilling the conditional exchangeability assumption. An argument for the score fulfilling this assumption is that it is constructed using estimates that have adjusted for additional covariates such as age and sex. On the other hand, the set of covariates differs per study, and the patients’ risk factor history is left unadjusted for. With regards to the consistency assumption, it is left unspoken what this hypothetical ‘prevention’ looks like, which makes it hard to argue whether it can estimate prevention potential or not. However, we must keep in mind that it is not directly relevant whether the assumptions are exactly true or not. Instead, it is the danger that the degree of wrongness in these assumptions may skew the results away from the truth.

Rather than relying on these causal assumptions, another option is to empirically verify whether the LIBRA score can predict the effect of an intervention. To empirically verify whether LIBRA estimates the full prevention potential is impossible, because we cannot verify whether the best possible intervention to prevent dementia was applied in a study. Instead, it could be verified whether LIBRA estimates the effect of interventions applied in real studies. In one study, no relationship between the LIBRA score and the effect of a multidomain lifestyle intervention has been found.^33^ This could be an indication that LIBRA does not in fact estimate the prevention potential of individuals for this specific intervention. It should be further explored by analysis of intervention studies whether LIBRA truly predicts the effect of an intervention on lifestyle.

### Surrogate outcome

Six articles have utilized, or plan to utilize LIBRA as a surrogate outcome measure for dementia in their intervention study.^12,64,66,68,73,79^ Nineteen articles have mentioned that the LIBRA score may be utilized as a surrogate outcome measure. The use of LIBRA as a surrogate outcome has the advantage that researchers do not have to wait for their participants to obtain their dementia diagnosis, which may take many years.

However, this application of LIBRA runs into similar issues as using it as a measure of ‘prevention potential’. When utilizing LIBRA as a surrogate outcome measure, changes in LIBRA score (i.e., changes in the risk factors that are a part of LIBRA) are implicitly equated to a change in dementia risk. Of the six papers that utilize the LIBRA score as a surrogate outcome, one paper mentions that a change in LIBRA might not reflect a change in dementia risk.^12^ Similarly to the use of the score as ‘prevention potential’, empirical evidence can help prove that LIBRA is an accurate indicator of dementia risk as a response to interventions. This could be done by comparing the dementia risk reduction in randomized intervention trials with the change in the dementia risk score.

Among the included publications that used LIBRA as a surrogate outcome we identified four different intervention trials: LETHE,^64^ Brain Bootcamp,^66,68^ PRIMA-Brain^73^ and AgeWell.^79^ All of these trials included physical activity, cognitive activity and diet as domains in the intervention. These domains are also risk factors included in the LIBRA score. One trial even specifically focused on LIBRA risk factors, by using an app intervention that included a topic for each LIBRA risk factor.^73^ However, since LIBRA is not proven to be reflective of changes in dementia risk as a response to intervening on the risk factors, perhaps it is better utilized as a measure of adherence to the study protocol, rather than an outcome measure in and of itself.^70^

### Targeting subpopulations

14 papers in total mentioned the usage of LIBRA to target which populations would benefit most from an intervention. Herein lies the implicit assumption that interventions would be most effective in the population with the highest LIBRA risk score. There is some merit to this assumption. Namely, if the (change in) risk of dementia follows a multiplicative scale, and if reducing a risk factor changes your relative risk by a fixed amount, then it follows that people with a higher baseline risk would benefit more in terms of absolute risk reduction. However, no significant effect modification of LIBRA on the effect of a multidomain lifestyle intervention has been found so far. In the PreDIVA study as well as the FINGER study, no effect modification by LIBRA had been found.^33,53^ Deckers et al. are also planning to study heterogeneity of treatment effect with respect to baseline LIBRA score in the FINGER-NL study.^34^

Similarly, eight articles have mentioned that LIBRA could potentially be utilized as an inclusion criterion for an RCT. While no inclusion criterion can be ‘wrong’, the choice of only including people with a sufficiently high LIBRA score suggests that these are the people where a significant effect of the intervention may be found. This again assumes that a higher baseline LIBRA would indicate a higher prevention potential. In our included literature, three articles have actually utilized LIBRA as an inclusion criterion.^34,73,78^ These studies required the presence of one or two modifiable risk factors, which is likely chosen to make an intervention possible at all.

### Awareness

Ten of the studies in our sample were what we classified as ‘awareness studies’, where it was assessed how aware individuals (clinicians or non-clinicians) were of the different risk factors in LIBRA or where the risk score was used in a study or campaign to increase awareness for dementia prevention.^22,23,27,43,45,50,55,71,73,74^ While awareness of the relation between lifestyle factors and dementia is important, the added benefit of the LIBRA score is not always clear in these studies. In several cases, only the dementia risk factors present in the LIBRA index were used, in which case more recent information might have been available from the Lancet commission^2^ or the World Health Organization. In other cases, the LIBRA index was used as a tool for participants to compute their own ‘prevention potential’. For this application, we again argue that this implies that reduction of these risk factors in LIBRA would reduce your dementia risk by an amount proportionate to the LIBRA score.

### Supporting clinician decisions

Ten papers have suggested that the LIBRA may be utilized to ‘support clinician decision making in practice’, and in seven papers it was mentioned that the LIBRA score could be used to guide risk modification.^15,47^ While examining the risk factors of an individual can definitely be informative, and signifies which risk factors an individual could improve, this application again assumes that LIBRA predicts the potential effect of intervening on an individual. The LIBRA score is validated as an indicator of dementia risk, and may therefore indeed be utilized to give an indication of that. However, care must be taken when utilizing LIBRA to give advice about which risk factors are most important to change.

## Discussion

### Key findings

We examined the range of applications of the LIBRA score reported in the scientific literature and critically assessed their suitability. We found substantial variation in how the score is used, the extent to which underlying assumptions are acknowledged, and the degree of empirical support available for different applications. These findings show that several applications of the LIBRA score require stronger empirical support and more explicit consideration of their underlying assumptions. By clarifying current applications and encouraging improvements in future use, we hope to contribute to the growing demand for tools that can effectively guide dementia prevention.

In our scoping review, we identified 66 publications that utilize the LIBRA risk score in some way. We have separately scored studied applications and mentioned applications of the LIBRA score in eight different categories: dementia prediction, association cognition, association other, adjust for lifestyle, surrogate outcome, targeting subgroups, inclusion criteria and awareness. Additionally, we scored the use of LIBRA for the selection of variables and mentions of the LIBRA as a measure of ‘prevention potential’ and as a tool for supporting clinician decisions in practice. Many of the included studies are motivated by the need for dementia reduction strategies. While examining associations involving LIBRA could help to find new possible pathways for dementia prevention, caution is warranted when interpreting such results as causal.

### Risk prediction versus risk intervention

It has become clear that the LIBRA score has become a popular measure for research into associations related to dementia. Indeed, many associations have been found between LIBRA and dementia, cognitive outcomes and others. However, it is not always clear what the objective of each study is, beyond an investigation of an association of LIBRA, or how the results should be interpreted. Some studies do mention more transparent objectives, such as the goal of exploring possible underlying pathways^32,59^ or examining differences in dementia risk in certain groups.^31,42^ For some associations, the LIBRA score is used as a surrogate measure for dementia risk, without always mentioning the limitations of this approach. Many other studies mostly validate the association of LIBRA with dementia and cognitive outcomes in different populations, often finding different values for the predictive ability of the LIBRA score. Although, if we are looking for the best model for dementia risk prediction, it would be better to also include non-modifiable risk factors. For many other applications, we are not directly interested in risk prediction, but are actually wondering who to apply our intervention on, or how effective a potential intervention would be. In these cases, a model that predicts risk of dementia does not necessarily provide us with our answer, but we should use a model that predicts a high potential effect of an intervention. Perhaps it would be more informative to validate dementia risk scores in other ways than solely the prediction of dementia risk, for example by evaluating how well the risk scores predict the effect of a lifestyle intervention.

The LIBRA score is neither fully optimized for prediction, nor does it explicitly claim to estimate a causal effect. The result of this is that the LIBRA is not as predictive of dementia risk as it could be (by including non-modifiable factors such as age), and that the underlying causal assumptions remain unacknowledged. Although risk prediction is a much easier task than the prediction of a causal effect, we should not shy away from admitting that we are actually interested in this causal effect, and simply choose to do prediction instead. If we acknowledge our interest in the causal effect, then we can also be honest about the assumptions that are necessary, and whether we have sufficient evidence that the effect we are estimating comes close to a causal effect. On the other hand, if we refuse to be honest about the fact that we are actually interested in the causal effect of an intervention, then we omit talking about the underlying assumptions that are necessary for our actual goals. We encourage developers of dementia risk scores to be honest about wanting to estimate causal effects, so that we directly address the questions that we are truly interested in.

### Lifestyle drift

Several studies used the LIBRA score as a motivational tool or as part of an awareness campaign. While creating awareness about the possible impact of lifestyle on dementia is important, this approach could contribute to a phenomenon called ‘lifestyle drift’, where risk reduction strategies shift their focus from population to individual. LIBRA specifically focuses on risk factors that could be modified by individual actions. In the development of the extension of LIBRA^62^ several risk factors, such as air pollution and formal education, were excluded because they were not deemed suitable for reduction on an individual level. A population level approach for dementia prevention, which aims to change societal conditions in a way that makes healthy choices the default or easiest option,^82^ could be more effective in the long run. Several studies by Röhr et al.^58–60^ that were included in our review also mentioned this phenomenon, noting that ‘lifestyle interventions should be part of a much more holistic public health strategy for risk reduction and prevention of cognitive decline and dementia’. This approach might even be able to prevent the occurrence of a risk factor entirely, which could be more effective than intervening later in life. Individual intervention efforts can still play a role, especially to mitigate the risk for groups were these risk factors are already present and for newly identified risk factors.^2,62^

### Relation to other risk scores

In our scoping review, we have only examined the applications of the LIBRA risk score, and do not examine the applications and mentioned applications of other risk scores such as CAIDE and ANU-ADRI. However, CAIDE and ANU-ADRI are constructed quite similarly, and have similar evidence as to their proposed applications.^3,83^ While these risk scores are not explicitly designed with the goal of predicting dementia prevention, they have also been proposed for causal applications. The CAIDE score has been utilized as a surrogate outcome measure.^12^ The ANU-ADRI dementia risk score has similarly been utilized as a primary and secondary outcome for intervention trials.^84^ For this reason, our conclusions about the applications of LIBRA may also be extrapolated to CAIDE and ANU-ADRI. These risk scores, similarly to the LIBRA score, have been constructed using observational data, and are optimized for observational tasks. Therefore, caution must be taken before assuming that these risk scores may also be utilized for causal tasks, such as the prediction of the effect of a hypothetical intervention or use as a surrogate outcome measure. More evidence is necessary to confirm that change in these risk scores truly reflects a change in dementia risk.

### Future research

For future research, we recommend that it is assessed per proposed application of dementia risk scores whether there is enough evidence for this application. For the applications where the evidence is lacking, in particular those assuming that LIBRA predicts the effectiveness of (hypothetical) interventions, we recommend that ongoing lifestyle intervention trials, such as the FINGER-NL study, critically evaluate whether the change in LIBRA is truly related to an actual change in dementia risk.

Secondly, researchers should continue to evaluate the effects of interventions on each individual risk factor on changes in dementia risk. This is important because if some LIBRA risk factors are not related to changes in dementia risk, then those risk factors should not be used in LIBRA's calculation for applications such as prevention potential estimation and use as a surrogate outcome. Furthermore, if the main goal is to estimate prevention potential, then the weights of the LIBRA score should be based on values from intervention studies rather than prospective cohort studies.

Finally, regardless of whether the score was constructed from intervention studies or prospective cohort studies, we recommend that the possible pathways between risk factors be acknowledged. For example, physical activity might impact the BMI, which again might impact the risk of type 2 diabetes. Therefore, an intervention on physical activity may impact dementia through other risk factors as well. This issue should be addressed in two different ways. Firstly, interactions between variables should be included into the LIBRA calculation, because this would result in a different assigned value to an intervention on physical activity on someone with or without type 2 diabetes. Secondly, when estimating the effect of changing a risk factor using intervention studies as per our previous advice, then the changes in other risk factors need to be adjusted for, rather than only the other factors at baseline.

### Conclusion

Importantly, we do not recommend against utilizing dementia risk scores in ways other than dementia prediction. Clearly, there is a strong demand for measures that can help us select which individuals to include in trials, which intervention to apply on these individuals, and which people would benefit most from an intervention. Despite limited evidence available to support these applications, dementia risk scores currently appear to be the best tools we have at our disposal. In terms of how we talk about dementia risk scores including LIBRA, we recommend that complete information is provided about what the score is utilized for, and what evidence there is that supports this application. This enables other researchers to critically evaluate the way the score is applied, and to draw their own conclusions.
